# Impact of AferBio® on quality of life and chemotherapy toxicity in advanced lung cancer patients (AFERBIO study): protocol study for a phase II randomized controlled trial

**DOI:** 10.1186/s12885-019-5599-z

**Published:** 2019-04-25

**Authors:** Daniel D’Almeida Preto, Mariana Toledo Baston, Camilla Centurion Geraige, Sarah Bertazzi Augusto, Marco Antonio de Oliveira, Augusto Elias Mamere, Gustavo Dix Junqueira Pinto, Josiane Mourão Dias, Pedro Rafael Martins De Marchi, Bianca Sakamoto Ribeiro Paiva, Carlos Eduardo Paiva

**Affiliations:** 1 Health-Related Quality of Life Research Group (GPQual), Learning and Research Institute, Barretos Cancer Hospital, Barretos, São Paulo Brazil; 2 Department of Clinical Oncology, Barretos Cancer Hospital, Rua Antenor Duarte Vilella, 1331, Bairro Dr Paulo Prata, Barretos, SP Brazil; 3 Faculty of Nutrition, Faculdade Barretos, Barretos, São Paulo Brazil; 4 Center for Epidemiology and Biostatistics, Barretos Cancer Hospital, Barretos, São Paulo Brazil; 5 Radiology Department, Barretos Cancer Hospital, Barretos, São Paulo Brazil

**Keywords:** Neoplasms, Lung cancer, Palliative care, Quality of life, Nutrition and drug-related side effects

## Abstract

**Background:**

Lung cancer patients undergoing palliative chemotherapy exhibit many symptoms related to the disease, such as adverse events and infectious complications during treatment, which impacts directly their health-related quality of life (HRQOL). Nutritional status is a relevant aspect among advanced cancer patients under palliative care and food supplementation has the potential to reduce treatment-related adverse effects and improve the nutritional status. The product named AferBio® is a fermented supplement that has been described as able to provide some benefits, including the capacity to potentiate the effects of anticancer drugs, by promoting the reduction of side effects and ultimately improving HRQOL.

**Methods/design:**

A Phase II double-blind placebo-controlled randomized clinical trial to assess the use of food supplementation with AferBio® in Stage IIIB or IV non-small cell lung cancer (NSCLC) patients beginning a second-line palliative mono-chemotherapy. The primary goal is to compare HRQOL scores between the arms of the study over time. The ten first patients included in the present study will undergo an AferBio®toxicity-testing (non-randomized phase). If no significant toxicity is found, the study will move on to the randomized phase. All patients will be randomized in blocks at a 1:1 ratio using the online tool REDCap. ECOG-PS (0–1 versus 2) criteria will be used for stratification. All patients included in the trial will be evaluated at baseline and *at each chemotherapy cycle*. Each evaluation will include the following: HRQOL (EORTC QLQ-C30, LC13 and IQualiV-Lung), ECOG-PS, anthropometric measurements, clinical and laboratory toxicity assessment and response evaluation.

**Discussion:**

During palliative systemic therapy in advanced cancer patients, one of the main goals is the improvement and maintenance of HRQOL, which can be negatively affected by cancer symptoms, cancer- or treatment-related psychosocial difficulties, and chemotherapy toxicity. Thus, much research has been dedicated to the development of new and more effective and/or less toxic cancer therapies. The present study is justified by the testing of a novel food supplement that may reduce some toxicities, thus, having a potential positive impact on the HRQOL of lung cancer patients. The product in question (AferBio®) is already available for sale in Brazil, but has not yet been fully tested in cancer patients.

**Trial registration:**

This Trial was registered on March 19, 2018 with ClinicalTrials.gov, NCT03469063. Protocol version: 2.0 from March 26, 2018. Trial status: Patient enrollment in the study began in April, 2018.

## Background

According to the World Health Organization (WHO), lung cancer is the most commonly diagnosed cancer in Brazil and worldwide, and it is by far the major cause of cancer-related deaths [1, 2]. Non-small cell lung cancer (NSCLC) accounts for approximately 80% of all lung cancers [3]. It develops silently and is nearly asymptomatic in its early stages [4]. In developed countries almost 60% of all cases are diagnosed in late stages of the disease, already displaying distant metastases or when curative treatment is no longer feasible [5]. The estimated 5-year survival rate for this cancer is no more than 4.5% [6].

Advancements in medicine have promoted reduced mortality rates and increased life expectancy. Some treatment regimens with palliative chemotherapy in daily practice have increased overall survival, reduced symptoms related to the disease and improved performance status, however causing side effects [3, 7–9]. In this scenario, the discussion of quality of life (QOL) becomes of paramount importance because we must offer an increased overall survival but with the best-possible quality [10]. WHO has determined QOL as *“an individual’s perception of their position in life in the context of the culture and value system in which they live and in relation to their goals, expectations, standards and concerns”* [11]. This is a complex and multidimensional concept that includes many domains, such as physical, emotional and social well-being, and functional capacity [12].

Evaluation of the nutritional status during the management of patients with advanced cancer in palliative care is a relevant aspect. Generally, cancer patients are at a high nutritional risk, which must be identified in a timely manner to establish a suitable nutritional plan to reduce hospital stay, infectious complications and morbidity rates, improving health–related quality of life (HRQOL) [13, 14]. Nutritional supplementation has the potential to reduce treatment-related adverse effects, improve and stabilize the nutritional status and, if necessary, increase caloric intake [14]. In this context, malnutrition and cachexia are the main consequences for cancer patients, unintentional weight loss is common and may intensify during chemotherapy due to its side effects [15, 16]. In patients with lung cancer, nutritional status is inversely correlated with pain, anxiety and depression scores, highlighting the need for early supportive psychotherapy or interventions focused on nutrition aspects [17].

The product AferBio® (“Fermented food for life”), created by a Brazilian biochemist and pharmacist, is a fermented supplement in powder form obtained through biotechnological processes during which substances such as β-glucans are synthesized and enzyme hydrolysis breaks down proteins into peptides and amino acids and complex carbohydrates into simple ones, thus favoring digestibility and absorption. It is a prebiotic and a source of β-[1–6] glucans, amino acids (among which 8 are essential), vitamin B12, and selenium, which is an antioxidant that exhibits some benefits, including the capacity to potentiate the effects of anticancer drugs, which promotes the reduction of side effects and ultimately improves QOL [18].

AferBio® is already available in the market, but has not yet been fully tested in cancer patients. Therefore, the goal of the present phase II randomized controlled trial is to assess the impact that this novel food supplement has on HRQOL and on the reduction of treatment-related complications in patients with advanced lung cancer that are beginning a palliative chemotherapy treatment. The trial will test the assumption that patients receiving AferBio should have fewer adverse events and fewer infectious complications during treatment compared with patients receiving placebo, which would lead to a better quality of life and fewer treatment delays. If the intervention proves successful, we will have a new supportive product to be used as an adjuvant to chemotherapy in patients with advanced lung cancer.

## Methods/design

### Ethical aspects

The present study was developed according to the norms of Resolution CNS 466/12 (Brazilian National Health Council) and was approved by the Research Ethics Committee of the Barretos Cancer Hospital (n° 2.395.325). This trial is registered with ClinicalTrials.gov, NCT03469063. All individuals complying with the inclusion criteria will be invited to participate in the study by a trained research coordinator from the Researcher Support Cencer of the Barretos Cancer Hospital. Each participant will voluntarily sign an Informed Consent Form (ICF). This study will be carried out in accordance with International Conference on Harmonization Good Clinical Practice (ICH-GCP). Every amendment in the research protocol will be first submitted to Research Ethics Committee approval. To avoid breaking data confidentiality only some of the authors (CEP, BSRP, and MAO) will have access to the final study database, which will be typed into online REDCap with restrictive access passwords.

### Study design

This is a Phase II double-blind placebo-controlled randomized clinical trial with two arms, intervention and control group conducted in only one center (Barretos Cancer Hospital [BCH], Brazil). The first 10 patients initiating chemotherapy with docetaxel infused every 21 days will undergo toxicity-testing for AferBio® during the three months of treatment. Table 1 illustrates some reported toxicities in previous studies with docetaxel, as well as the maximum tolerated toxicity determined a priori for the non-randomized phase of the study. If no criteria of unacceptable toxicity are met, then the study will move to the randomized phase. Furthermore, indicators of toxicity of the experimental product will also be assessed during the randomized phase of the study.

**Table 1:** Expected toxicity and threshold limit values for the first 10 patients receiving standard mono-chemotherapy with docetaxel 75 mg/m2 each 21 days [7]

Toxicity	Grade of toxicity		Threshold limit value	
	Any	≥ 3	Any	≥ 3
Anemia	90%	10.6%	–	> 3 of 10
Neutropenia	–	76%	–	–
Febrile Neutropenia	–	11–25%	–	> 4 of 10
Thrombocytopenia	8–14%	1%	> 3 of 10	> 1 of 10
AST	5%	–	> 2 of 10	> 1 of 10
ALT	5%	–	> 2 of 10	> 1 of 10
Bilirubin	6–9%	2%	> 2 of 10	> 1 of 10
Creatinine	5%	< 1	> 1 of 10	–
Urea	5%	< 1	> 1 of 10	–
Constipation	11.7%	0.4%	> 3 of 10	–
Diarrhea	36.4–39%	1.8–5%	> 5 of 10	> 2 of 10
Mucositis	25.5%	1.8%	> 4 of 10	–
Nausea	36.4–39%	3.6–4%	> 5 of 10	> 2 of 10
Vomiting	22–23.6%	3–3.6%	> 4 of 10	> 2 of 10
Asthenia	54.5%	18.2%	> 7 of 10	> 4 of 10
Infection	30.9%	5.5%	> 4 of 10	> 2 of 10

Patients will be randomly assigned (1:1) by researchers, who will have no clinical contact with patients; researchers will use the online tool REDCap [19]. After assignment to interventions, trial participants, care providers, outcome assessors and data analysts will be blinded; only the professionals responsible for the randomization process and a pharmacist responsible for the control of research products (who will not have direct contact with the participants) will be unblinded. The status of the Eastern Cooperative Group Performance Status (ECOG-PS) will be used for stratification (0–1 versus 2). All patients will receive chemotherapy according to the Department of Thoracic Oncology and will comply with the institute protocols regarding dose and procedure in cases of toxicity.

The primary goal is to compare HRQOL scores between the arms of the study over time. Secondary goal is to assess AferBio® safety and adherence; compare between the arms of the study the incidence of any given toxicity ≥ grade 3 (jointly and individually); worsening-free survival of 20% of HRQOL scores: number of days that the treatment was delayed due to toxicity; infection or worse performance status; compare the dose intensity (in mg/m2/week); dose-reduction rates (≥20%) and calculate dose reduction-free survival; number of hospitalizations; number of infections (any grade), use of systemic anti-microbial, number of chemotherapy cycles with addition of granulocyte colony-stimulating factor (G-CSF) and the incidence of febrile neutropenia; compare worsening-free survival of the ECOG-PS (> 1 point); anthropometric measurements between the arms of the study; compare response rates and progression-free survival.

### Patients

Eligible patients will be required to be at least 18 years old and less than 75 years, and to have a 0 to 2 ECOG-PS, with a pathologically confirmed TNM Stage IIIB or IV NSCLC beginning any line of palliative mono-chemotherapy from the second-line. They must have adequate hematological (total neutrophil count ≥1500/μL, platelet count ≥100.000/μL and hemoglobin ≥9 g/dL), liver (serum bilirubin ≤1.5 × ULN, aspartate aminotransferase (AST), alanine transaminase (ALT), alkaline phosphatase ≤1.5 × ULN), and kidney (serum creatinine ≤1.5 × ULN based on the Cockcroft−Gault equation) functions; absence of any emotional family-related, sociological, or geographic condition that can potentially hamper adherence to the study protocol and the follow-up schedule (as per investigator).

Patients will be excluded from the study at the researcher’s discretion if there is tube feeding, gastrostomy or jejunostomy; uncontrollable vomiting; sexually active women in reproductive age, except for those who underwent surgical sterilization; intestinal obstruction or sub-obstruction; known allergy to any of the components of the investigational product; malabsorption syndrome or other condition that could interfere with enteric absorption; history of inflammation of the small or large intestine, previous or currently active (such as Crohn’s disease or ulcerative colitis); chronic diarrhea of any cause; diagnosis of any chronic disease that, in the researcher’s opinion, will interfere in their participation in the study; known diagnosis of HIV infection; diagnosis of any chronic disease that changes the immune system and significantly increases the risk of infection; the need to use G-CSF already in the first chemotherapy cycle; severe neuropsychiatric disease that prevents the patient from completing the study questionnaires.

### Procedures

Before initiating chemotherapy, patients will receive sachets containing AferBio® or placebo. They will be instructed by a nutritionist or nurse concerning how to ingest the product.

The fermented powder supplement will be provided by the company AferBio Bioalimentos, Ltd. (Santa Barbara D’Oeste, São Paulo, Brazil, ANVISA No. 6.04980–1). Each sachet will contain 10 g of the investigational product or placebo. Patients will be instructed to dissolve the contents of the sachet in 150 mL to 200 mL of soy milk, fruit juice, or iced tea and to shake it until homogenization is complete. A measuring cup (with 150 mL and 200 mL marks) will be provided to study participants. Patients will drink the product once a day for seven days and then twice a day continuously (for a total of three months). Participants will also be instructed to not discard used sachets, which shall be handed back to the research team during the return visits in order to be counted. The use of another nutritional supplement will not be allowed during the study.

### Data collection and instruments

**Fig. 1:**
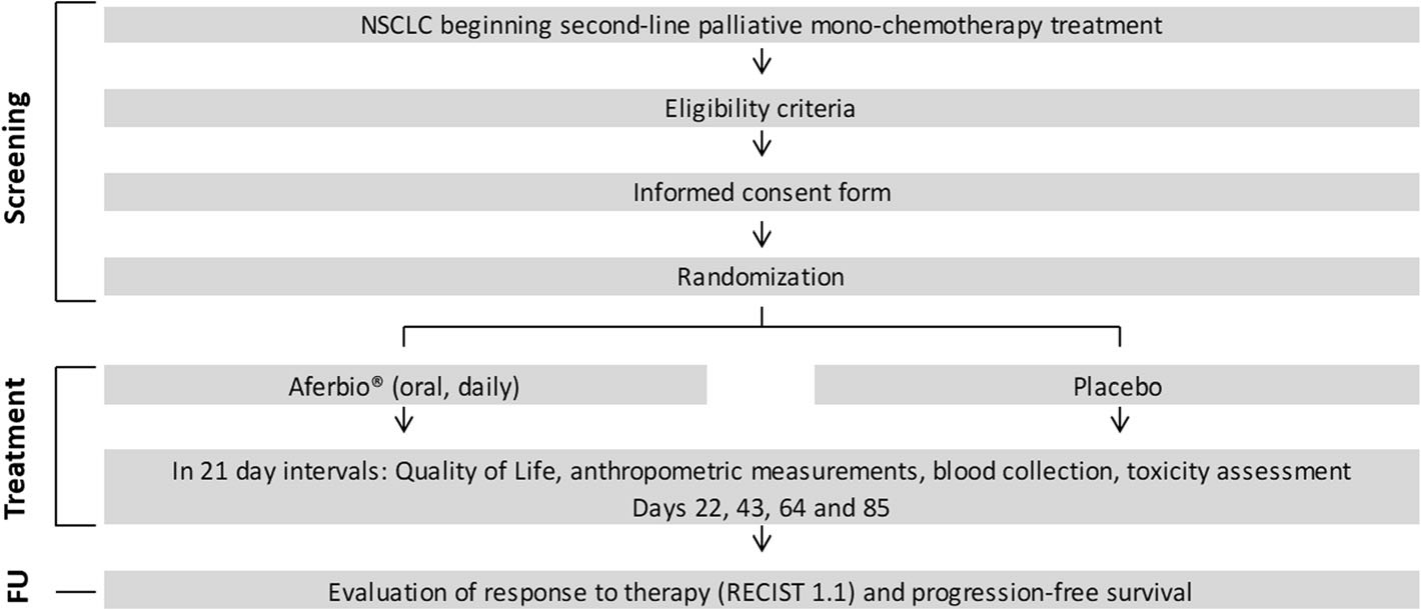
Schematic overview of the study design. In the non-randomized phase of the study, the same evaluations will be carried out, but all participants will receive the investigational product

All participating patients, both in the non-randomized and in the randomized phase of the trial, will be evaluated at baseline and at each chemotherapy cycle (Fig. 1). All evaluations will be performed while patients are in the hospital for medical visits and/or anticancer treatment (chemotherapy). No patient will be called to the hospital exclusively for study-related procedures.

Researchers will apply 3 questionnaires to measure HRQOL: The European Organization for Research and Treatment of Cancer Quality of Life Core Questionnaire with 30 items (EORTC QLQ C30), composed of 30 items that comprise functional, symptom, global health scales and financial impacts [20], validated in 2010 in Brazil [21, 22]. The European Organization for Research and Treatment of Cancer Quality of Life Questionnaire Lung Cancer Module (EORTC LC13), Brazil by Brabo/Brazil by Brabo et al [23, 24] is a specific supplementary module for lung cancer patients receiving chemotherapy or radiotherapy, composed of 13 questions addressing typical symptoms of lung cancer and treatment-related side effects [25]. The Instrument of Evaluation of the Quality of Life with 24 items for evaluation of patients with lung cancer (IQualiV-Lung) is a version of IqualiV-OG-21, a generic instrument for the evaluation of HRQOL, developed at the Barretos Cancer Hospital and validated in a Brazilian multicenter study [26]. Three specific items were included in IqualiV-OG-21 to assess the HRQOL in lung cancer patients (dyspnea, cough and hemoptysis), totaling 24 items, which was renamed as IQualiV-Lung.

The ECOG-PS scale will assess how the disease affects the daily living abilities of patients [27].

The following Anthropometric measurements will be used to assess the patient nutritional status during the length of the study: body mass index (BMI) [28–30], triceps skinfold (TSF) thickness, weight, height [31], adductor pollicis muscle thickness (APMT) on the dominant side [32, 33].

The clinical and laboratorial side effects will be classified according to its severity by the Common Terminology Criteria for Adverse Events (CTCAE), a grading scale ranging from 1 (mild) to 5 (death related to adverse event) [34].

Response Evaluation Criteria in Solid Tumors (RECIST), version 1.1, will guide the evaluation of the oncological status related to the response to chemotherapy as complete response (CR), partial response (PR), stable disease (SD) and progressive disease (PD) [35, 36].

In cases of PD and consequently modification in the anticancer treatment, the participant will be withdrawn from the study. In this case, a final assessment of HRQL and anthropometric measurements will be conducted.

### Statistical analyses

Enrolment of 94 participants (47 in each arm) was planned to have a power (1– β error) of 80% and alpha error of 0.05, when estimating an effect size of 0.25. Sample-size calculations were estimated with *Gpower* software v.3.0 (Heinrich-Heine-Universität, Düsseldorf, Germany) [37], considering a 20% loss during the study.

Demographic and clinical characteristics prior to treatment will be compared between the arms of the study with the Student’s t test (for continuous variables) or chi-square test (for categorical variables). The normality of the data distribution will be assessed with the Shapiro-Wilk test [38] combined with an evaluation of data distribution patterns.

All HRQOL scores and anthropometric measurements will be compared over time using a linear mixed model, considering the treatment group and time as fixed effects and patients as a random effect. Thus, the effect of the interaction time versus arm and the effects of the arm alone or time alone will be assessed, and the variability will be controlled by the effect of the patient. Multiple imputation of missing data will be used.

The number of patients with toxicity ≥ grade 3, hospitalization, infections, use of antimicrobials, febrile neutropenia, adherence to the investigational product/placebo, dose reduction above 20%, and clinical benefit rates will be compared between groups by the chi-square test (or Fisher’s exact test). Clinical benefit rate will be considered the percentage of patients who have achieved CR, PR and SD.

The total days of treatment delay, dose intensity, anthropometric measurements, and the number of chemotherapy cycles with G-CSF will be compared between groups at the end of the study using the Student’s t test (or Mann-Whitney U test, depending on the data normality).

Event-free survival times (worsening of HRQOL > 20%, worsening by > 1 point in the ECOG-PS score, and ≥ 20% dose reduction) will be estimated by Kaplan-Meier curves, which will be compared with the log-rank test.

All analyses will be conducted as intention-to-treat analyses. Statistical significance will be considered at *p* < 0.05. Statistical analyses will be performed with IBM SPSS Statistics for Windows, version 21.0 (IBM Corp., Armonk, N.Y., USA).

### Monitoring committee

An independent monitoring committee that consists of staff from the Research Support Center of BCH will monitor the collection and analysis of the study data. Meetings of the Monitoring Committee were planned to occur at least every six months.

### Interim data analysis

An interim analysis is planned to be conducted after the inclusion of the first 10 participants with complete data (non-randomized phase). If no criteria of unacceptable toxicity are met (as detailed in Table 1), then the study will move to the randomized phase. In case of doubts about the toxicity of the investigational product after 10 participants, an addendum will be sent to the Ethics Committee requesting approval for inclusion of another 10 patients in the non-randomized phase. The Monitoring Committee will participate in the interpretation of the results. The principal investigator (CEP) will be the main responsible for the decision to stop or continue the study. Sponsor will not participate in this decision process.

## Discussion

Studies have been conducted to assess the likely effects of AferBio® in animals and humans. One study was performed using rats fed with the product for 90 days. There were no signs of systemic toxicity, and in general, organs were preserved [39]. Another study assessed the cytotoxic activity of an AferBio® extract in several human tumor cell lines that were cultivated and replicated under sterile conditions. In those analyses, a concentration of 25 μg/mL inhibited growth in colon and lung cell lines [40]. Additionally, a promising AferBio anti-inflammatory activity was verified in a study using a rat inflammatory model. In this study, wistar rats were fed the AferBio product (900 mg / kg / day) for 30 days before the induction of inflammation and for 6 more days; significant effects in both acute and chronic models of inflammation were observed [41].

Patients infected with the human immunodeficiency virus (HIV) eventually develop acquired immunodeficiency syndrome (AIDS), which renders them susceptible to infections and tumor development. Commonly, HIV patients exhibit unintentional weight loss, functional changes in the gastrointestinal system, and increased nutritional needs [42, 43]. A group of 17 HIV patients completed a QOL questionnaire that assessed physical, emotional, social, environmental, and health-related aspects, both before and after supplementation with AferBio®. Body mass index (BMI) was also compared before and after supplementation. In general, all participants exhibited improvements in HRQOL; BMI showed little change, which was considered to be satisfactory. Thus, supplementation with AferBio® can be used as an adjuvant in the treatment of these patients and also in maintaining their weight [43].

In another study, patients with chronic kidney disease on hemodialysis who suffered from constipation due to the side effects of a component of their drug therapy commonly prescribed by nephrologists, i.e., calcium carbonate, received supplementation with AferBio® to assess the impact of this high-fiber product on bowel transit. Patients who correctly followed the instructions reported improvements in bowel transit, which resulted in enhanced HRQOL. After interruption of the supplementation with AferBio®, patients reported recurrence of constipation [44].

Lung cancer patients receiving second-line palliative mono-chemotherapy exhibit many symptoms related to the disease, adverse events and infectious complications that impact directly in HRQOL. As the nutritional status is an important aspect among advanced cancer patients under palliative care, this study was designed to evaluate if a supplementation with AferBio®, which has not yet been fully tested in cancer patients, may improve the HRQOL, reduce treatment-related adverse effects and improve the nutritional status of the intervention group. The study was also designed to evaluate the toxicity of the product.

### Aknowledgements

We would like to thank the research coordinator Gabriela, from the Researcher Support Center of the Barretos Cancer Hospital fot her help in data collection.

## Abbreviations

ALT: Alanine transaminase
APMT: Adductor pollicis muscle thickness
AST: Aspartate aminotransferase
BCH: Barretos Cancer Hospital
BMI: Body mass index
CTCAE: Common Terminology Criteria for Adverse Events
ECOG-PS: Eastern Cooperative Group Performance Status
EORTC LC13: The European Organization for Research and Treatment of Cancer Quality of Life Questionnaire Lung Cancer
EORTC QLQ-C30: The European Organization for Research and Treatment of Cancer Quality of Life Core Questionnaire with 30 itens
G-CSF: Granulocyte colony-stimulating factor
HRQOL: Health-related quality of life
ICF: Informed Consent Form
ICH-GCP: International Conference on Harmonization Good Clinical Practice
IQualiV-Lung: The Instrument of Evaluation of the Quality of Life with 24 items for evaluation of patients with lung cancer
NSCLC: Non-small cell lung cancer
QOL: Quality of life
RECIST: Response Evaluation Criteria in Solid Tumors
TSF: Triceps skinfold
ULN: Upper limit of normal
WHO: World Health Organization
